# Violence and sexual risk taking reported by young people at Swedish youth clinics

**DOI:** 10.48101/ujms.v127.7823

**Published:** 2022-01-21

**Authors:** Sofia Hammarström, Siw Alehagen, Helena Kilander

**Affiliations:** a Region Västra Götaland, Knowledge Center for Sexual Health, Gothenburg, Sweden; b Division of Society and Health, Department of Health, Medicine and Caring Sciences, Linköping University, Linköping, Sweden; c Division of Nursing Sciences and Reproductive Health, Department of Health, Medicine and Caring Sciences, Linköping University, Linköping, Sweden; d Department of Obstetrics and Gynaecology, Eksjö Hospital, Jönköping, Sweden; e Jönköping Academy for Improvement of Health and Welfare, Jönköping University, Jönköping, Sweden; f Department of Women’s and Children’s Health, Karolinska Institutet, Sweden

**Keywords:** Gender identity, transgender youth, violence, risk taking, sexual health, re-victimisation, poly-victimisation, cumulative violence

## Abstract

**Background:**

Early identification of sexual risk taking and exposure to violence is fundamental when seeking to strengthen young people’s health. The purpose of this study was to study factors associated with sexual risk taking and ill health, as well as to study gender differences, and the associations amongst exposure to multiple forms of violence, sexual risk taking and ill health.

**Methods:**

This was a cross-sectional study based on data from 3,205 young people answering a questionnaire belonging to the Sexual health Identification Tool (SEXIT 2.0), during consultations at 12 youth clinics in Sweden. The analyses are based on descriptive statistics and nominal multiple regression analysis.

**Results:**

Male, transgender and non-binary youths reported significantly more events of sexual risk taking and ill health compared to women. Those who reported sexual initiation before the age of 15 (OR 2.87, CI 1.81–4.56), three or more sexual partners in the past 12 months (OR 2.68, CI 1.70–4.22) and to have ever experienced an unintended pregnancy (OR 2.29, CI 1.32–3.97) were more than twice as likely to report exposure to physical, emotional and sexual violence. Transgender, non-binary youths and women were more exposed to multiple violence (OR 3.68, 13.50) compared to men.

**Conclusions:**

Transgender and non-binary youths are exposed to significantly more violence compared to women and men. Experiences of sexual risk taking and ill health demonstrated strong associations with exposure to multiple violence.

## Introduction

Globally, young people defined as the 10–24 years age group are disproportionally burdened by sexual and reproductive health problems, such as sexually transmitted infections (STIs), unintended pregnancies (1) and exposure to sexual and gender-based violence (2). These experiences not only affect health and well-being directly, but, as experiences during a critical development phase in life, may also lead to long-term negative effects (3). Early detection of young people in need of care or at risk of sexual ill health or violence victimisation is important in order to provide adequate care and preventive measures (4).

Sexual risk taking is commonly defined as risking an unintended pregnancy and/or STI by not using a condom or other contraception. Previous experience of chlamydia infection and unintended pregnancy can also be considered risk factors, as they are associated with repeated chlamydia infections (5, 6) or unintended pregnancies (7). Other factors found to be associated with sexual risk taking are substance use (8–10), early sexual initiation (<15 years) (11) and multiple sexual partners (5, 12). Additionally, some experiences may increase the risk of unprotected sex through limited or absent ability to negotiate safer sex, such as transactional sex and violence victimisation (13).

Concerning violence, the consequences on health include depression (14, 15), anxiety (15), post-traumatic stress disorder (14, 15), substance abuse (14, 16), self-injury (14), suicidal behaviour (17) and poorer self-rated health (16), amongst others.

In a Swedish study, the prevalence of violence victimisation was shown to be 10 times more common amongst youths than amongst adult samples (18). Most studies have focused on a single type of violence (physical/emotional/sexual) or a specific situation, i.e. dating violence, bullying or child sexual abuse. However, violence exposure is rarely an isolated event (8), and physical, emotional and sexual violence often co-occur (18, 19). Young people (20, 21) as well as adults (22) with multiple violence exposure report more ill health than those suffering from repeated exposure to only one type. There are gender patterns where men report more physical abuse, whilst women report more exposure to emotional and sexual abuse (18).

However, knowledge of the impact of multiple forms of violence on the lives of young people is limited, and the connection to sexual risk taking and ill health has been little explored. Blom et al. (23) found an increased risk for experience of pregnancy, chlamydia, early sexual initiation and ≥3 sexual partners in both men and women exposed to multiple forms of violence. As their measure of multiple violence included sexual violence, a question warranting exploration is whether the elevated risks are predominantly related to being exposed to multiple forms of violence, or if they are primarily attributed to sexual violence victimisation.

The associations between exposure to multiple forms of violence and sexual risk taking have, to our knowledge, not been studied amongst transgender and non-binary youths. In this study, we use ‘transgender and non-binary’ to refer to young people whose sex assigned at birth does not match their current gender identity. Studies suggest that transgender and non-binary youths are more exposed to discrimination and violence victimisation than their cis-gendered peers (24–26), resulting in both poor mental health (25–27) and negative effects on sexual health, such as HIV (28), avoidance of sexual situations out of fear, abandoning safer sex practices at times of mental ill health and lack of access to sexual health services (29).

Swedish studies suggest that female youth clinic visitors are more exposed to violence (31%) (19) than young women in general (21%) (23). This makes youth clinics a suitable arena not only to study relationships amongst violence victimisation, sexual risk taking and sexual ill health but also to clinically address the associated health needs.

Sweden has a nationwide system of youth clinics, providing a wide range of services related to sexual and reproductive health and rights (SRHR) and psychosocial health concerns, and accessible to young people between the ages of 12 and 25 (30). Most visitors are women (89%), often explained by a focus on contraceptive services (30). Data on youth clinic utilisation are lacking. One study found that 16% of youths had visited a youth clinic in the past 3 months (31). In recent years, many youth clinics have introduced a tool, SEXIT (SEXual health Identification Tool), aiming to systematically address sexual risk taking, ill health and violence exposure with all visitors, through a questionnaire (32). This provides a unique opportunity to study a large sample of youth clinic visitors from different parts of Sweden, regarding multiple forms of violence and sexual risk taking. The aim of this study was to study factors associated with sexual risk taking and ill health, as well as to study gender differences, and the associations amongst exposure to multiple forms of violence, sexual risk taking and ill health.

## Materials and methods

### Design/setting

This study is based on quantitative results from a questionnaire belonging to the SEXIT 2.0 (32), within a framework of quality improvement (QI) and collaboration between youth clinics and the Knowledge Centre for Sexual Health in the southwest of Sweden. The QI involved implementation of SEXIT in clinical practice.

### Procedure and participants

To reach a range of youth clinics covering different socio demographics in Sweden, youth clinics were invited from participating counties with traditionally different statistics in abortion and STIs. Information about the study was sent to the heads of 12 youth clinics participating in the QI, and all agreed to participate.

Youth clinic visitors were offered the opportunity to answer the questionnaire in connection to their index visit. After the visitor had answered the questionnaire, a healthcare professional (HCP) had a dialogue with the visitor about the responses to make a risk assessment regarding the visitor’s needs for support or treatment. In addition, the HCPs were trained to have a dialogue about sexuality and to use the handbook, which apart from suggesting follow-up questions also clarifies which answers indicate risk exposure and recommends relevant measures. Data from the questionnaires were evenly collected over 6 months by all youth clinics in the period 2017–2019.

### SEXIT 2.0

SEXIT was developed to facilitate the identification of young people exposed to, or at risk of, sexual ill health and includes three components: 1) a training of HCPs, 2) a questionnaire for visitors and 3) a handbook for HCPs to support the dialogue and risk assessment. The questionnaire includes 20 items concerning age, gender identity, sexual orientation, living situation, alcohol and drug uses, experiences of violence, age at time of first sexual initiation and number of sexual partners during the past 12 months. Furthermore, the questionnaire includes items about the experience of unintended pregnancy and STIs, use of contraception and protection against STIs, sex for reimbursement and experiences of coercing/forcing someone sexually.

The development of SEXIT 2.0 was based on a previous pilot study (32) where the instrument was developed, validated and pilot-tested at three Swedish youth-clinics. Based on the results of the pilot implementation, the authors further developed SEXIT by including items regarding physical and emotional violence, witnessing violence, protection against STI and pregnancy and experience of STIs other than chlamydia, resulting in version 2.0. The content validity of SEXIT 2.0 was established by an expert panel. Most of the variables in the questionnaire have been previously validated (32).

The questionnaires did not gather social security numbers, names or contact details. Each questionnaire had a unique code to enable digital processing, but the code was not connected to an individual. As no personal data were collected in the study, the Swedish Ethical Review Authority found no need for ethical approval (#: 2019/03628).

### Variable definitions

The gender identity alternatives ‘transgender’, ‘other gender’ and ‘do not wish to categorise myself’ were summarised into the category ‘Transgender and non-binary’.

Variables associated with sexual risk taking and ill health according to previous research were dichotomised as follows: alcohol use two or more times a month, ever drug use (8–10), sexual initiation before 15 years of age (11), three or more sexual partners during the past 12 months (12, 23), ever experience of own or partner’s unintended pregnancy (1), ever experience of chlamydia (5, 6), ever experience of sex for reimbursement and ever experience of coercing/forcing someone sexually (9).

The response option ‘don’t know *and yes*’ for the items regarding unintended pregnancy, chlamydia and coercing/forcing someone sexually was considered as sexual risk taking or ill health.

Experiences of multiple violence were defined as experiences of more than one category of violence, including physical, emotional and sexual violence. Physical violence was defined as being beaten or hurt. Emotional violence was defined as being threatened, harassed or bullied. Sexual violence was defined as being victim of coerced/forced sex, i.e. unwanted sexual experiences including vaginal/oral/anal sex or touching someone’s genitals.

### Data analysis

Of the 3,576 questionnaires, 16 were excluded due to missing values of gender and/or age. Visitors who answered that they had had not initiated sex were asked to finish the questionnaire before the questions on sexual history started. These participants (*n* = 355) were excluded from the analysis, resulting in a total of 3,205 questionnaires. The internal attrition rate in individual items was below 3%.

Data grouped by gender were analysed by using descriptive statistics. Categorical data were analysed with Chi-square test. If more than 20% of the cells have an expected value <5, we used Fischer’s exact test when calculating *P*-values. The Mann–Whitney U test is used in ordinal scales with three or more alternatives when calculating *P*-values. We analysed gender differences in dichotomised variables associated with sexual risk taking as well as violence victimisation.

Multinomial regression analysis was used to yield a model predicting the most important explanatory variables associated with the seven different groups of violence victimisation. The variables alcohol and drug use, sexual initiation before 15 years of age, three or more sexual partners during the past 12 months, to have ever experienced their own or partner’s unintended pregnancy and to have ever had chlamydia were found significant in the model fitting of the multinomial regression analysis and therefore included. Seven different groups of violence were entered as dependent variables, and the independent variable no violence was entered as a reference. In the next step, gender was included for analysing gender differences with men entered as reference. Age as a continuous variable was included as a covariate in the analysis. The multinomial regression analysis indicated an overall classification of 67%, and the results are presented as odds ratio (OR) with 95% confidence interval (CI). The variables regarding sex for reimbursement as well as experiences of coercing/forcing someone sexually were not included in the analysis due to limited data. Statistical analyses were performed using IBM SPSS statistics version 22.0.

## Results

### Demographics, sexual risk taking and ill health

Around 80% of the participants were heterosexual women aged 18–25 years old. Some gender differences could be observed: being bisexual was more common amongst participants in the transgender group and amongst women compared to men. Across genders, most participants reported living with their parents. However, more women reported living with a partner, compared to the other gender groups (Table 1).

**Table 1:** Demographics, sexual history and analysis of gender differences.

	Women *n* (%)	Men *n* (%)	Transgender *n* (%)	Women vs. men/Women vs. trans/Men vs. trans *P*-value
Total number of participants, *n* = 3,205	2,682 (84)	453 (14)	70 (2)	
Age, years (*n* = 3,168)				
13–17	659 (25)	80 (18)	29 (42)	
18–25	1,994 (75)	366 (82)	40 (58)	
Mean age (SD)	19 (SD 2.49)	20 (SD 2.33)	18 (SD 2.6)	
Sexual orientation (*n* = 3,178)				<0.001/<0.001/<0.001^b^
Heterosexual	2,334 (88)	412 (91.5)	45 (64)	
Homosexual	13 (0.5)	22 (5)	6 (9)	
Bisexual	270 (10)	15 (3)	16 (23)	
None of the categories	40 (1.5)	2 (0.5)	3 (4)	
Living arrangement (*n* = 3,168)				<0.001/0.001/<0.001^b^
Alone	488 (18.4)	101 (22.9)	9 (12.9)	
With parents	1,568 (59)	274 (62)	43 (61.4)	
In foster family/institution	10 (0.4)	2 (0.5)	3 (4.3)	
With friends	141 (5.3)	24 (5.4)	3 (4.3)	
With partner	377 (14.2)	33 (7.5)	6 (8.6)	
Other arrangement	72 (2.7)	8 (1.8)	6 (8.6)	
Use of alcohol (*n* = 3,159)				<0.001/ns/0.006^c^
≥4 times/week	18 (0.7)	10 (2.2)	1	
2–3 times/week	271 (10.3)	68 (15.1)	6 (8.7)	
2–4 times/month	1,090 (41.3)	195 (43.2)	24 (34.8)	
≤1 time/month	1,003 (38)	139 (31.8)	26 (37.7)	
Never	257 (9.7)	39 (8.6)	12 (17.4)	
Median (IQR)	3 (1)	3 (1)	4 (1)	
Use of drugs (*n* = 3,179)				<0.001/0.040/0.004^c^
Yes, during the past 30 days	75 (2.8)	40 (9)	2 (2.9)	
Yes, during the past 12 months	179 (6.7)	69 (15.5)	11 (15.7)	
Yes, more than 12 months ago	260 (9.8)	75 (16.8)	7 (10)	
Never	2,149 (80.7)	262 (58.7)	50 (71.4)	
Median (IQR)	4 (0)	4 (1)	4 (1)	
Sexual debut <15 (*n* = 3,113)				ns/0.003/ns^b^
No	2,149 (82)	342 (79)	47 (72)	
Yes	464 (18)	93 (21)	18 (28)	
No. of sexual partners during the past 12 months (*n* = 3,156)				<0.001/ns/0.002^c^
0	55 (2)	19 (4)	6 (9)	
1–2	1,760 (66)	179 (41)	39 (57)	
3–4	443 (17)	110 (25)	12 (17)	
5–10	336 (13)	102 (23)	10 (14)	
11–20	48 (2)	24 (6)	2 (3)	
≥21	6 (0)	5 (1)	0 (0)	
Median (IQR)	2 (1)	2 (2)	2 (1)	
Ever had chlamydia infection (*n* = 3,162)				<0.001/ns/ns^c^
Yes, during the past 12 months	131 (4.9)	18 (4.1)	5 (7)	
Yes, more than a year ago	199 (7.5)	29 (6.6)	4 (6)	
Never	2,176 (82)	320 (72.6)	52 (75)	
Don’t know	146 (5.5)	74 (16.8)	8 (12)	
Median (IQR)	3 (0)	3 (0)	3 (0)	
Use of condom against STI (*n* = 3,122)				ns/ns/ns^c^
Always	518 (20)	56 (13)	17 (26)	
Often	693 (26)	157 (36)	14 (21)	
Seldom	715 (27)	138 (32)	24 (36)	
Never	696 (27)	83 (19)	11 (17)	
Median (IQR)	3 (2)	3 (1)	3 (2)	
Ever experienced unintended pregnancy, own or partner (*n* = 3,168)				<0.001/ns/ns^c^
Yes	181 (7)	39 (9)	8 (12)	
No	2,429 (91)	371 (84)	56 (84)	
Don’t know	49 (2)	32 (7)	3 (4)	
Median (IQR)	2 (0)	2 (0)	2 (0)	
Use of protection against unintended pregnancy (*n* = 3,117)				<0.001/0.002/ns^c^
Always	1,924 (73)	230 (53)	40 (59)	
Often	418 (16)	128 (30)	10 (15)	
Seldom	153 (6)	30 (7)	9 (13)	
Never	124 (5)	42 (10)	9 (13)	
Median (IQR)	1 (1)	1 (1)	1 (2)	

^a^Transgender (trans) includes identification as transgender, non-binary and those not willing to categorise themselves by gender.

b Use of Pearson’s *X*^2^.

c Use of Mann–Whitney test.

In items with ≤5, use of Fischer’s exact test. Missing values 26–92 per item. SD = standard deviation, IQR = interquartile range.

Half of all participants reported using alcohol twice a month or more. Experience of ever having used drugs differed from 19 to 41% between gender groups (Table 2). Men reported significantly more use of alcohol and drugs compared to both women and the transgender group (Tables 1 and 2).

**Table 2:** Frequency and gender differences in reported variables associated with sexual risk taking.

Variables	Women	Men	Transgender^a^	Women vs. men/Women vs. trans^a^/Men vs. trans^a^

	*n* = 2,682	*n* = 453	*n* = 70	

	%	%	%	*P*-value
Alcohol use ≥2 times a month	53	60	45	0.001/ns/0.010
Ever used drugs	19	41	29	<0.001/0.054/0.040
≥3 sexual partners during past 12 months	35	55	35	<0.001/ns/0.002
Sexual initiation <15 years of age	18	21	28	ns/0.030/ns
Ever had chlamydia	18	27	25	<0.001/ns/ns
Ever had unintended pregnancyb	9	16	16	<0.001/0.020/ns
Received imbursement for sex	1	1	6	ns/0.003/0.001
Given imbursement for sex	0	3	0	<0.001/ns/ns
Coercing/forcing someone sexuallyc	3	6	3	0.001/ns/ns

Use of Pearson’s *X*^2^ test. Use of Fischer’s exact test in items ≤5.

a Transgender (trans) includes identification as transgender, non-binary and those not willing to categorise themselves by gender.

^b^Includes own or partner’s experience of unintended pregnancy. ^c^Includes feeling that the sex was against the partner’s will, through pressure.

The reported age of first sexual initiation ranged from 10 to 24 years of age (mean 16, SD 1.7). Furthermore, 18% of all participants reported sexual initiation before the age of 15 years, and this was more common amongst the participants in the transgender group. The number of sexual partners in the past 12 months varied between 0 and 38 (mean 2.7, SD 3.1). Men reported significantly more sexual partners during the past 12 months (Table 1), and more often three or more partners compared to women and the transgender group (55% vs 35%, *P* = 0.002) (Table 2). Men and transgendered participants reported experience of unintended pregnancy to a greater extent compared to women (16% vs 9%, *P* = 0.020) (Table 2). Furthermore, men and transgendered participants reported never having used protection against unintended pregnancy more often, compared to women (10–13% vs 5%, *P* = 0.002) (Table 1).

Amongst reported variables associated with sexual risk and ill health, the three most commonly reported variables across all gender groups were alcohol use twice a month or more, three or more sexual partners during the past 12 months, and ever having used drugs (Table 2). Experience of ever having been reimbursed for sex was reported to the same extent by men and women (1%) but was more common amongst participants in the transgender group (6%) (Table 2). Furthermore, experience of ever having reimbursed someone else for sex was only reported by men (3%), and coercing/forcing someone sexually was more common amongst men (6% vs 3%) (Table 2).

### Experience of emotional, physical, sexual violence and gender differences

In total, experience of emotional violence was the most frequently reported type of violence victimisation (21%), compared to sexual violence (15%) and physical violence (12%) (Table 3). Some gender differences were observed; physical violence was considerably more reported by men and participants in the transgender group compared with women (23–24% vs 10%, *P* = 0.001). Both emotional violence and sexual violence were more common amongst participants in the transgender group and women compared to men (5–6% vs 1%, *P* = 0.008) (Table 3) (OR 9.80, 12.70) (Table 4). Experiences of both physical, emotional and sexual violence were more reported by participants in the transgender group (9%) in comparison to both men and women (9% vs 2–4%, *P* = 0.020) (Table 3) (OR 13.50) (Table 4).

**Table 3:** Reported experiences of violence and analysis of gender differences.

Violence victimisation (responding participants)	Women *n* (%)	Men *n* (%)	Transgender^a^ *n* (%)	Women vs. men/Women vs. trans^a^/Men vs. trans^a^ *P*-value
	2,682 (84)	453 (14)	70 (2)	
Physical violence (*n* = 3,205)				<0.001/<0.001/ns
No	2,421 (90)	351 (77)	53 (76)	
Yes	261 (10)	102 (23)	17 (24)	
Emotional violence (*n* = 3,205)				<0.001/0.008/<0.001
No	2,086 (78)	392 (86)	45 (64)	
Yes	596 (22)	61 (14)	25 (36)	
Sexual violence (*n* = 3,137)				<0.001/0.005/<0.001
No	2,176 (83)	411 (93)	51 (74)	
Yes	450 (17)	31 (7)	18 (26)	
Physical and emotional violence (*n* = 3,205)				0.010/0.007/ns
No	2,600 (97)	428 (94)	63 (90)	
Yes	82 (3)	25 (6)	7 (10)	
Physical violence and sexual violence (*n* = 3,205)				ns/ns/ns
No	2,661 (99)	447 (99)	69 (99)	
Yes	21 (1)	6 (1)	1 (1)	
Emotional violence and sexual violence (*n* = 3,205)				<0.001/ns/0.008
No	2,560 (95)	450 (99)	66 (94)	
Yes	122 (5)	3 (1)	4 (6)	
Physical, emotional and sexual violence (*n* = 3,205)				0.030/0.020/0.008
No	2,585 (96)	444 (98)	64 (91)	
Yes	97 (4)	9 (2)	6 (9)	

Use of Pearson’s *X*^2^. In items with ≤5, use of Fischer’s exact test.

a Transgender (trans) includes identification as transgender, non-binary and those not willing to categorise themselves by gender.

**Table 4:** Odds ratio (OR) for associations between violence victimisation, gender and variables associated with sexual risk taking and ill health.

Method = Enter no violence as reference In gender, enter men as reference	B	S.E.	Wald	P-value	OR	CI (95%)
Physical violence (*n* = 126)						
Age	0.03	0.04	0.63	0.426	0.96	0.88–1.05
Alcohol use ≥2 times a month	0.06	0.22	0.09	0.761	1.06	0.69–1.64
Ever used drugs	0.82	0.22	13.66	<0.001*	2.29	1.47–3.55
≥3 sexual partners during past 12 months	0.31	0.21	2.08	0.148	1.36	0.89–2.08
Sexual initiation <15 years of age	0.75	0.23	10.48	<0.001*	2.12	1.34–3.35
Ever had chlamydia	0.32	0.22	2.06	0.151	1.38	0.88–2.16
Ever had unintended pregnancy^a^	0.33	0.29	1.29	0.255	1.40	0.78–2.51
Women	−1.58	0.21	54.02	<0.001*	0.20	0.13–0.31
Transgender	−0.56	0.63	0.77	0.378	0.57	0.16–0.19
Emotional violence (*n* = 327)						
Age	0.07	0.02	7.24	0.007*	1.07	1.02–1.13
Alcohol use ≥2 times a month	−0.02	0.13	0.04	0.838	0.97	0.74–1.27
Ever used drugs	0.57	0.15	13.19	<0.001*	1.77	1.30–2.41
≥3 sexual partners during past 12 months	0.04	0.14	0.11	0.735	1.05	0.79–1.38
Sexual initiation <15 years of age	0.79	0.15	26.44	<0.001*	2.20	1.63–2.98
Ever had chlamydia	−0.34	0.17	3.77	0.052	0.73	0.52–1.03
Ever had unintended pregnancy^a^	0.22	0.22	1.04	0.306	1.25	0.81–1.93
Women	0.82	0.23	12.88	<0.001*	2.28	1.45–3.58
Transgender	1.20	0.48	6.30	0.012*	3.34	1.30–8.57
Sexual violence (*n* = 230)						
Age	0.07	0.03	4.90	0.027*	1.07	1.01–1.15
Alcohol use ≥2 times a month	0.21	0.17	1.55	0.213	1.23	0.88–1.72
Ever used drugs	0.74	0.17	17.49	<0.001*	2.10	1.48–2.98
≥3 sexual partners during past 12 months	0.36	0.16	5.02	0.025*	1.44	1.04–1.99
Sexual initiation <15 years of age	0.72	0.18	15.25	<0.001*	2.07	1.43–2.98
Ever had chlamydia	−0.17	0.19	0.73	0.390	0.84	0.57–1.24
Ever had unintended pregnancy^a^	0.18	0.26	0.51	0.471	1.20	0.72–2.02
Women	1.46	0.33	18.65	<0.001*	4.30	2.21–8.34
Transgender	2.07	0.56	13.64	<0.001*	7.99	2.65–24.06
Physical and emotional violence (*n* = 114)						
Age	−0.01	0.04	0.08	0.774	0.98	0.90–1.07
Alcohol use ≥2 times a month	−0.52	0.22	5.26	0.022*	0.59	0.37–0.92
Ever used drugs	1.28	0.23	29.61	<0.001*	3.61	2.27–5.74
≥3 sexual partners during past 12 months	0.23	0.23	1.01	0.315	1.26	0.80–1.98
Sexual initiation <15 years of age	0.43	0.25	2.94	0.086	1.54	0.94–2.55
Ever had chlamydia	0.01	0.25	0.002	0.965	1.01	0.61–1.66
Ever had unintended pregnancy^a^	1.23	0.26	22.26	<0.001*	3.44	2.06–5.76
Women	0.00	0.28	0.00	1.000	1.00	0.57–1.74
Transgender	1.36	0.52	6.69	0.010*	3.91	1.39–11.01
Physical violence and sexual violence (*n* = 28)						
Age	0.08	0.09	0.80	0.369	1.08	0.90–1.29
Alcohol use ≥2 times a month	−0.19	0.45	0.17	0.672	0.82	0.34–2.00
Ever used drugs	1.72	0.44	15.25	<0.001*	5.59	2.35–13.25
≥3 sexual partners during past 12 months	1.04	0.44	5.54	0.019*	2.85	1.19–6.84
Sexual initiation <15 years of age	0.77	0.45	2.93	0.087	2.17	0.89–5.29
Ever had chlamydia	0.17	0.44	0.15	0.691	1.17	0.50–2.82
Ever had unintended pregnancy^a^	1.19	0.47	6.47	0.011*	3.30	1.31–8.30
Women	0.44	0.52	0.70	0.400	1.560	0.55–4.38
Transgender	1.40	1.14	1.50	0.220	4.05	0.43–37.93
Emotional violence and sexual violence (*n* = 129)						
Age	0.02	0.04	0.26	0.610	1.02	0.94–1.11
Alcohol use ≥2 times a month	0.08	0.21	0.14	0.708	0.92	0.60–1.41
Ever used drugs	1.15	0.21	28.52	<0.001*	3.17	2.07–4.86
≥3 sexual partners during past 12 months	0.32	0.21	2.32	0.127	1.38	0.91–2.09
Sexual initiation <15 years of age	0.79	0.22	12.25	<0.001*	2.21	1.41–3.44
Ever had chlamydia	0.57	0.21	6.82	0.009*	1.76	1.15–2.71
Ever had unintended pregnancy^a^	0.12	0.32	0.15	0.697	1.13	0.60–2.14
Women	2.28	0.59	14.72	<0.001*	9.80	3.05–31.47
Transgender	2.54	0.85	8.90	0.003*	12.70	2.39–67.44
Physical, emotional violence and sexual violence (*n* = 112)						
Age	0.07	0.04	2.31	0.128	1.07	0.98–1.17
Alcohol use ≥2 times a month	−0.83	0.23	12.52	<0.001*	0.43	0.27–0.68
Ever used drugs	1.55	0.23	44.61	<0.001*	4.74	3.00–7.49
≥3 sexual partners during past 12 months	0.98	0.23	18.04	<0.001*	2.68	1.70–4.22
Sexual initiation <15 years of age	1.05	0.23	20.10	<0.001*	2.87	1.81–4.56
Ever had chlamydia	0.25	0.24	1.11	0.292	1.29	0.80–2.08
Ever had unintended pregnancy^a^	0.83	0.28	8.28	0.003*	2.29	1.32–3.97
Women	1.30	0.39	11.04	0.001*	3.68	1.70–7.96
Transgender	2.60	0.60	18.52	<0.001*	13.50	4.12–44.19

B = B-coefficient. S.E. = Std Error. Wald = Wald-test. OR = Odds ratio. CI = Confidence interval. A = includes own or partner’s experience of unintended pregnancy.

* P=< 0.05.

### Multiple forms of violence and the relationship to variables associated with sexual risk taking and ill health

**Figure 1:**
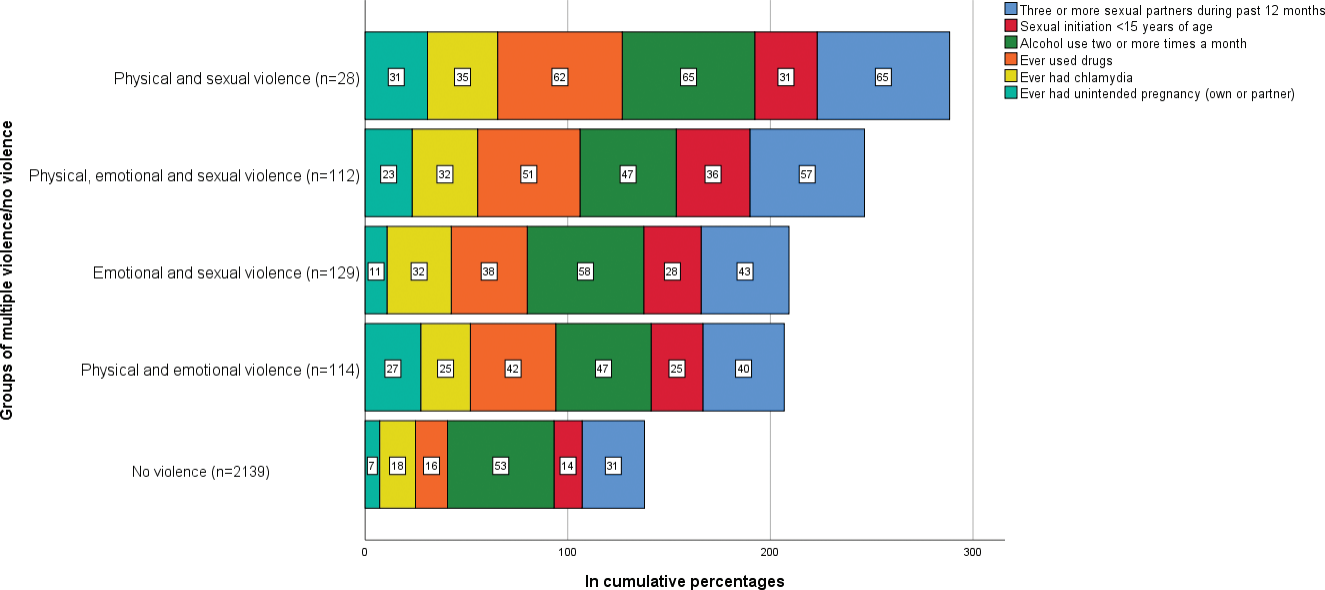
Reported variables associated with sexual risk taking amongst visitors reporting experiences of multiple violence versus no violence.

Participants reporting both physical, emotional and sexual violence, or the combination of both physical and sexual violence reported higher proportions of variables associated with sexual risk taking, compared to participants reporting other combinations of multiple violence or no violence. Ever having used drugs, alcohol use twice a month or more, and three or more sexual partners during the past 12 months were the most commonly reported variables associated with sexual risk taking amongst visitors reporting experiences of multiple violence (Figure 1).

Regarding violence victimisation, higher age increased the odds of participants reporting sexual or emotional violence (OR 1.07). Most combinations of violence resulted in raised odds ratios for drug use (OR 1.77–5.59) and sexual initiation before the age of 15 (OR 2.07–2.87) (Table 4). Participants reporting both physical, emotional and sexual violence were more than twice as likely to report three or more sexual partners in the past 12 months, experience of unintended pregnancy and sexual initiation before the age of 15 years. Participants reporting physical violence or multiple violence were two to four times as likely to report ever having used drugs (OR 2.29, 4.74). In addition, participants reporting experiences of both physical, emotional and sexual violence or physical violence and sexual violence had considerably higher ORs of three or more sexual partners in the past 12 months (OR 2.68–2.85) and experience of unintended pregnancy (OR 2.29–3.30) (Table 4).

## Discussion

Our results show that young people reported multiple forms of violence and sexual risk taking during consultations at youth clinics, showing them to be a vulnerable group of young people and reflecting gender inequalities in health.

Those reporting experiences associated with sexual risk taking and ill health were more likely to report violence victimisation. Participants reporting experiences of taking drugs were more than four times as likely to report both physical, emotional and sexual violence. Those who reported ≥3 sexual partners during past 12 months, early sexual initiation and unintended pregnancy were twice as likely to experience physical, emotional and sexual violence. Taken together, experience of both physical, emotional and sexual violence was overrepresented amongst transgender, non-binary youth and women. Their reported experiences of violence demonstrated strong associations with sexual risk taking. This demonstrates the urgent need to identify the vulnerable groups when visiting youth clinics, especially since previous research has identified lower self-rated health amongst young people who report both physical, emotional and sexual violence (33).

Through our research design, including different combinations of violence exposure, we can confirm that previously demonstrated associations between sexual risk taking and multiple forms of violence (23) were consistent also in the combination of only physical and emotional violence. This indicates that sexual risk taking is an important factor for experiencing multiple forms of violence amongst young people. However, our study was cross-sectional and cannot establish causality regarding when violence and sexual risk taking occurred. A likely hypothesis is that vulnerable adolescents have the double disadvantage of being at risk for both violence and sexual ill health. It has been suggested that polyvictimisation in adolescents to a large degree explains trauma symptoms, including sexual concerns (3). There is also evidence, suggesting that childhood abuse is associated with later high-risk sexual behaviours (34, 35) and re-victimisation (36), causing a dangerous circle of risk taking and victimisation.

Regarding gender inequalities in health, this study shows that the male and transgendered visitors were markedly more at risk than their female counterparts in almost every variable measured. Men demonstrated higher alcohol consumption, more drug use and more sexual partners than other genders. Men also reported more experience of unintended pregnancy, previous chlamydia infection, of giving reimbursement for sex and of coercing/forcing someone sexually, compared to women. Except for drug use, these gender differences are not in accordance with studies of youth in the general population in Sweden (23, 37), where gender differences are small and sometimes reversed. For example, previous chlamydia infection and unintended pregnancy are usually reported by a majority of women (23, 37). In summary, our results could illustrate a gender aspect that men visiting youth clinics are a selection of more sexually risk-taking men. Previous studies have shown that men visiting youth clinics receive less counselling on protection in connection to STI tests and abortion, compared to women (37). This is particularly troublesome with our results in mind and a major impediment to gender-equal sexual health.

It is worth noting that the associations between variables related to sexual risk taking and violence victimisations demonstrated in Table 4, to a great extent reflect the female population that was in majority.

In comparison to all genders, the transgender and non-binary group particularly stand out regarding early sexual initiation and experience of receiving reimbursement for sex. Our results reveal that the transgendered and non-binary participants suffered from high rates of exposure to multiple forms of violence, including physical, emotional and sexual violence, compared to other genders. This confirms and extends previous studies (24–26) by adding their vulnerability to multiple forms of violence.

It is a complex process to compare results from different studies as definitions, age groups and data collection procedures often differ. In comparison to studies of young people in the general population (23), the youth clinic population was found to be exposed to violence to a similar degree (33). Although our sample is not random, it is intended to be representative for Swedish youth clinics in general, and our results are comparable with other studies conducted in the Swedish youth clinic setting concerning demographic aspects such as gender (30, 33), age (8) and sexual orientation (38). However, the prevalence of physical, emotional and sexual violence observed in our study was lower than in another Swedish study using anonymous questionnaires in the youth clinic setting. The difference could be explained by the difference in response rates between the studies but could also be because our study used responses provided during consultation and not anonymously. Responses delivered during consultation and not anonymously are likely to be underestimated due to the sensitive nature of the questions and the risk of social desirability (39). On the other hand, a strength is that the design provides valuable information on what young people are prepared to discuss with HCPs.

A strength of our study is the relatively large sample size compared to the sparse research on young people (13–25 years of age). Furthermore, most studies on multiple violence have been conducted either on children (20, 21) or in adult populations (22).

SEXIT must be used systematically with every visitor, but as we lack information on visitors who were never offered the questionnaire or who chose not to respond, we cannot calculate the external attrition rate and there is a risk of selection bias. On the other hand, a strength of our study is the low internal attrition rate in individual items.

Concerning the variables, despite the item of alcohol consumption being designed primarily for clinical purposes and perhaps too rough, it still yielded similar results as studies using AUDIT-C in the youth clinic setting (10). A strength of our study is the collection and reporting of data on non-binary and transgendered people, often excluded in research explained by small numbers, but sometimes also on ignorance when collecting and reporting data (40). It is worth noting that the gender inequalities in our results may be underestimated as the item of gender identity may not capture individuals with a transgender history who are currently identifying as a man or woman. Furthermore, our analysis did not take sexuality into consideration, despite it being confirmed in many studies that homo and bisexual individuals are more exposed to violence than heterosexuals (19, 41). Lastly, SEXIT does not account for number of victimisation events within each form of violence, which can be of importance when measuring victimisation (3).

### Implications and future studies

The associations found between sexual risk taking and experience of multiple forms of violence, and the urgent health needs found in the youth clinic population have implications for both clinical work and research. If only a single form of violence is considered in research, a misinterpretation of the association between sexual risk taking and violence is likely. In the clinical context, the association between sexual risk taking and violence victimisation suggests that screening for multiple forms of violence and sexual risk taking should be offered to all visitors in order to identify young people in need of care and support.

## Conclusions

Young people with experiences of sexual risk taking and ill health report significantly more multiple violence victimisation. Transgender and non-binary youths are exposed to considerably more violence compared to women and men.
